# Satisfaction with body weight among adolescents with excess weight: findings from a cross-sectional population-based study

**DOI:** 10.1590/1516-3180.2020.0007.R1.10062020

**Published:** 2020-09-18

**Authors:** Mariana Contiero San Martini, Daniela de Assumpção, Marilisa Berti de Azevedo Barros, Antonio de Azevedo Barros, Josiemer Mattei

**Affiliations:** I PhD. Nutritionist, Department of Pediatrics, Faculdade de Ciências Médicas da Universidade Estadual de Campinas (FCM-UNICAMP), Campinas (SP), Brazil.; II PhD. Nutritionist, Department of Pediatrics, Faculdade de Ciências Médicas da Universidade Estadual de Campinas (FCM-UNICAMP), Campinas (SP), Brazil.; III MD, PhD. Titular Professor, Department of Public Health, Faculdade de Ciências Médicas da Universidade Estadual de Campinas (FCM-UNICAMP), Campinas (SP), Brazil.; IV MD, PhD. Collaborating Associate Professor, Department of Pediatrics, Faculdade de Ciências Médicas da Universidade Estadual de Campinas (FCM-UNICAMP), Campinas (SP), Brazil.; V MPH, PhD. Associate Professor, Department of Nutrition, Harvard T. H. Chan School of Public Health, Harvard University, Boston (MA), United States.

**Keywords:** Adolescent, Overweight, Obesity, Body weight, Body satisfaction, Teenager, Young obese, Excess weight, Underestimate, Overestimate

## Abstract

**BACKGROUND:**

Individuals who are overweight or obese often underestimate their size, and they are less likely to consider their weight status to be a health problem and consequently to make lifestyle changes.

**OBJECTIVES:**

To estimate the proportion of satisfaction with weight among adolescents classified as overweight/obese, according to sociodemographic factors, morbidities and health-related behaviors.

**DESIGN AND SETTING:**

Cross-sectional population-based study conducted among adolescents aged 10 to 19 years in the city of Campinas (SP), Brazil.

**METHODS:**

The sample (n = 217) included participants with self-reported weight and height who were classified as overweight or obese, based on body mass index (BMI) according to age-specific cutoff points recommended by the World Health Organization. Participants whose answer to the question: “Would you like to gain or lose weight?” was “no” (i.e. no change) were deemed to be satisfied with their body weight. Odds ratios and 95% confidence intervals (95% CI) were calculated using logistic regression.

**RESULTS:**

The proportions of the respondents who were satisfied with their weight were 75.8% (95% CI: 65.3-83.9) among the overweight adolescents and 24.2% (95% CI: 16.1-34.7) among the obese adolescents (P < 0.01). Satisfaction was lower among individuals aged 15 to 19 years (versus 10 to 14 years), those born outside of Campinas (versus in Campinas), those with ≥ 8 household appliances (versus < 8), and those reporting ≥ two health complaints (versus none).

**CONCLUSIONS:**

More than half of the overweight adolescents and almost a quarter of the obese adolescents were satisfied with their weight. These results support the need for strategies for healthy weight management among Brazilian adolescents.

## INTRODUCTION

The prevalence of obesity among children is 5.0% (107.7 million) and 12.0% among adults (603.7 million), according to a study conducted in 195 countries. Between 1990 and 2015, most nations experienced continuous increases in prevalence.^1^ In 2010, overweight and obesity were estimated to contribute 3.4 million deaths, 3.8% of disability-adjusted life-years and 3.9% of years of life lost among adults worldwide.^2^

In Brazil, the projections of overweight and obesity are of particular concern. In the adult population (≥ 18 years), the prevalence of overweight increased from 30.9% to 33.2% and obesity from 11.9% to 17.5%, from 2006 to 2013, which reflected an average annual increases of 2.3% and 5.6% in the respective prevalences.^3^ Among Brazilian adolescents aged 10 to 19 years, in 2008/2009, 20.5% were classified as overweight and 4.9% as obese.^4^ A study on adolescents aged 13 to 17 years found that 23.7% and 7.8% were classified as overweight and obese, respectively, in 2015.^5^ In addition, it has been estimated that by 2022, 13.8% of boys and 7.8% of girls in Brazil will be classified as obese.^6^

The Brazilian government has continuously monitored the trends of overweight and obesity and has developed strategies to reduce the increasing prevalence, such as encouraging breastfeeding, promoting physical activity, supplying healthy foods in the school environment, regulating food advertising aimed at children and adolescents and restricting the marketing of food products high in salt, sugar and unhealthy fats.^6^ Given the reported increases in obesity among Brazilians, these government-sponsored strategies are possibly not having much effect with regard to reducing excess weight in the population.

It is common for adolescents to have distorted body perceptions.^7^ They experience multiple physical, emotional and sociocultural influences that can affect self-perception of body image, thus causing dissatisfaction and even a distorted view of the body's own shape.^8^ According to the National School Health Survey (PeNSE 2015), approximately 20.0% of adolescents (13 to 17 years old) perceived themselves to be fat or very fat.^5^ Another study revealed that 13.5% of boys who were overweight or obese wanted to gain weight, probably with the expectation of gaining muscle mass. Among girls, there was greater dissatisfaction with overweight and obesity.^9^

Individuals who are overweight or obese tend to distort their weight perception, often through underestimating the size of certain body parts. They are less likely to consider their weight status to be a health problem and consequently to make lifestyle changes.^7^^,^^10^ Additionally, distorted body perceptions are more often present among people who experienced gain of weight later in life than among those that have been obese since childhood.^7^ Studies that evaluate weight satisfaction among adolescents who are overweight/obese are rare, and it is important to identify the epidemiological profile of this group in order to develop health promotion actions.

## OBJECTIVE

The objective of this study was to estimate the proportion of satisfaction with weight among adolescents classified as overweight or obese according to sociodemographic factors, morbidities and health-related behaviors.

## METHODS

### Study population

Data from a cross-sectional population-based health survey known as ISACamp were used. In this survey, information from non-institutionalized individuals residing in the urban area of Campinas, Brazil, were collected. This area has a population of more than one million inhabitants, living within 100 kilometers from the state capital of São Paulo, Brazil. The data were collected from February 2008 to April 2009.

The sample of the survey was recruited by means of probabilistic sampling procedures, using cluster sampling, in two stages: census tract and household. In the first stage, 50 census tracts were drawn, with probability proportional to the number of households. The census tracts of the Brazilian Institute for Geography and Statistics (Instituto Brasileiro de Geografia e Estatística, IBGE) were used, based on the 2000 census. The households of the selected census tracts were visited to obtain an up-to-date list of addresses, considering the length of time that had elapsed since the last census (which was in the year 2000). In the second stage, households were randomly selected.

The survey population consisted of three age domains: adolescents (10 to 19 years), adults (20 to 59 years) and elderly people (60 years or over). Only adolescents were analyzed in this study. The sample size was 1,000 people in each age domain, considering the following: maximum variability for the frequency of the events studied (P = 0.50); 95% confidence intervals (z = 1.96); sampling error between 4% and 5%; and design effect equal to 2. The response rate was taken to be 80% and, therefore, the sample size was corrected to 1,250. To achieve the desired sample size, 2,150, 700 and 3,900 households were drawn for interviews with adolescents, adults and elderly people, respectively.

Data were collected through a survey structured into 14 thematic blocks, which had been tested in a pilot study. The survey was applied by trained and supervised interviewers. The thematic block on food habits contained questions regarding weight and height (self-reported), practices used for weight loss and frequency of food consumption.

This study was conducted in accordance with the guidelines laid down in the Declaration of Helsinki and all procedures involving research study participants were approved by the Research Ethics Committee of Universidade Estadual de Campinas (UNICAMP) on January 13, 2015 under protocol number 931.774. Written informed consent was obtained from all subjects.

### Study variables

Satisfaction with body weight was the dependent variable and was assessed through the question “*Would you like to gain or lose weight*?”, with three answer options: “no” (i.e. no change); “yes, I'd like to lose weight”; and “yes, I'd like to gain weight”. If the participants answered “yes” to this question, they were then asked how much they would like to weigh. If they wanted to lose weight, they were asked whether they were undertaking any practices to lose weight, and if so, this was probed using a list of practices.

The independent variables that were assessed included demographic and socioeconomic characteristics, health-related behaviors and morbidities.

The sociodemographic characteristics selected were sex, adolescent age group (categorized as 10-14 years versus 15-19 years), place of birth (Campinas versus other city/state), *per capita* household income (in monthly minimum wages), education level of household head (in years of schooling), number of appliances in the household (out of a total of 15 items) and having health insurance (yes or no).

Health-related behaviors included food consumption, weight loss practices and physical activity. The participants' weekly consumption of fruits, vegetables and soft drinks was assessed from a simple food frequency questionnaire, in which the following responses were possible: “every day”; “four to six days a week”; “one to three days a week”; “less than once a week”; or “less than once a month”. These were then grouped into “≥ 4 times a week” versus “< 4 times a week”. The respondents were asked whether they did any practices to lose weight (yes or no), and if so, what practices were followed, from among these options: “careful about food intake”; “diet”; or “exercise, sports or walking”. Their leisure physical activity practices were investigated. Adolescents aged 10-17 years who practiced at least 60 minutes of physical activity per day, at least five days a week, were classified as “active”. Adolescents aged 18-19 years who performed at least 150 minutes per week, for three days, were also classified as “active”. These age classifications for physical activity levels followed the definitions of the World Health Organization. Adolescents who practiced physical activity at lower levels than recommended were classified as “insufficiently active”. Those who did not do any kind of physical activity were classified as “inactive”.^11^

The participants were asked to self-report any chronic diseases that had been diagnosed by a physician or other health professional. These could include hypertension, diabetes, cancer, arthritis, osteoporosis, asthma, heart disease, cancer, tendonitis and circulation problems. Likewise, they were asked about any health complaints such as headache/migraine, allergy, emotional problems, dizziness/vertigo, insomnia and back pain.

Nutritional status was evaluated using body mass index (BMI), defined as weight (kg)/height^2^ (m). This was calculated from self-reported weight and height and was classified according to World Health Organization age-specific BMI cutoff points: underweight BMI < 3^rd^ percentile; normal weight BMI ≥ 3^rd^ percentile and ≤ 85^th^ percentile; overweight BMI > 85^th^ percentile and ≤ 97^th^ percentile; and obese BMI > 97^th^ percentile.^12^

### Statistical analysis

This analysis only included adolescents aged 10 to 19 years of both sexes with self-reported weight and height and who were classified as overweight or obese. Among the selected households in which adolescents were present, 14.8% were not included because the resident was absent or refused to provide information. Out of the 955 adolescents identified from the households that had been drawn, 31 refused to participate. Self-reported weight and height data were collected in relation to 820 individuals. Among these, 217 were classified as overweight (16.2%) or obese (10.2%) and were included in this analysis.

The proportion of the adolescents who were satisfied with their body weight according to the independent variables was estimated. Differences according to categories were tested using Pearson's chi-square test, considering a significance level of 5%. Odds ratios and 95% confidence intervals (95% CI) were calculated using logistic regression. The sociodemographic and morbidity variables with P < 0.20 in the bivariate analysis were added to the model and those that continued to present P < 0.05 were kept in the final model.

The interview data were entered into a database that had been prepared in the EpiData 3.1 software (EpiData Association, Odense, Denmark). The statistical analyses were done in the Stata 11.0 software (Stata Corp., College Station, USA), in the survey module, which allows analysis of complex sample data.

## RESULTS

Body weight satisfaction was higher among younger adolescents aged 10 to 14 years (84.3%), individuals who reported not having any chronic disease (91.3%), those without health complaints (53.1%) and those classified as overweight (75.8%). Greater desire to lose weight was noted among adolescents who had ≥ 8 appliances in their home (57.3% among those with 8-15 appliances and 33.9% among those with 16 or more), compared with those with < 8 appliances (8.8%) (P < 0.01) (Table 1).

**Table 1:** Proportions of satisfaction/dissatisfaction with weight among adolescents aged 10 to 19 years who were overweight or obese, according to sociodemographic and morbidity factors and nutritional status. ISACamp health survey, 2008/2009

		n	% (95% CI)			P-value*
			Satisfied	Wished to gain weight	Wished to lose weight	
**Sex**						
	Male	127	64.1 (54.7-72.5)	72.4 (20.0-96.5)	55.4 (45.4-64.9)	0.34
	Female	90	35.9 (27.5-45.3)	27.6 (3.5-80.0)	44.6 (35.1-54.6)	
**Age (years)**						
	10 to 14	147	84.3 (74.3-90.9)	75.9 (23.0-97.1)	57.5 (48.2-66.2)	**< 0.01**
	15 to 19	70	15.7 (9.1-25.7)	24.1 (2.9-76.9)	42.5 (33.7-51.7)	
**Place of birth**						
	Campinas	173	87.7 (76.2-94.1)	75.9 (23.0-97.1)	74.7 (61.9-84.2)	0.09
	Other town/state	44	12.3 (5.9-23.8)	24.1 (2.9-76.9)	25.3 (15.8-38.1)	
***Per capita* income (monthly minimum wages)**						
	< 0.5	58	32.5 (22.8-44.0)	24.1 (2.9-76.9)	23.0 (15.8-32.1)	0.15
	≥ 0.5 to < 1.5	111	52.4 (41.9-62.7)	24.1 (2.9-76.9)	50.2 (38.3-62.0)	
	≥ 1.5	48	15.0 (7.9-26.7)	51.7 (12.3-89.1)	26.8 (18.1-37.9)	
**Schooling of head of household (years)**						
	0 to 7	79	32.7 (19.2-49.8)	24.1 (2.9-76.9)	38.4 (28.0-50.0)	0.66
	8 or more	136	67.3 (50.2-80.8)	75.9 (23.0-97.1)	61.6 (50.0-72.0)	
**Number of appliances in the home**						
	0 to 7	42	35.8 (21.6-53.0)	0.0	8.8 (3.8-19.2)	**< 0.01**
	8 to 15	115	45.2 (31.4-59.7)	48.3 (10.8-87.7)	57.3 (47.4-66.6)	
	16 or more	60	19.0 (9.6-34.1)	51.7 (12.3-89.1)	33.9 (23.5-46.1)	
**Health insurance**						
	Yes	77	28.9 (18.7-41.9)	24.1 (2.9-76.9)	42.7 (28.3-58.3)	0.18
	No	137	71.1 (58.1-81.3)	75.9 (23.0-97.1)	57.3 (41.6-71.7)	
**Number of chronic diseases**						
	0	175	91.3 (81.4-96.2)	75.9 (23.0-97.1)	75.7 (67.0-82.7)	**0.02**
	1 or more	39	8.7 (3.8-18.6)	24.1 (2.9-77.0)	24.3 (17.3-33.0)	
**Number of health complaints**						
	0	89	53.1 (40.3-65.5)	0.0	34.5 (27.0-42.8)	**< 0.01**
	1	61	37.0 (27.7-47.5)	75.9 (23.0-97.1)	21.3 (14.8-29.7)	
	2 or more	67	9.8 (4.8-19.0)	24.1 (2.9-76.9)	44.2 (35.3-53.5)	
**BMI (kg/m^2^)**						
	Overweight	133	75.8 (65.3-83.9)	100.0**	51.5 (43.8-59.1)	**< 0.01**
	Obesity	84	24.2 (16.1-34.7)	0.0	48.5 (40.9-56.2)	

n = number of individuals in the unweighted sample; 95% CI = 95% confidence interval; BMI = body mass index.

* P-value from chi-square test;

** four overweight adolescents reported that they wished to gain weight.

Trying to lose weight was significantly associated with weight satisfaction/dissatisfaction (P < 0.01), such that more dissatisfied adolescents reported trying to lose weight. More than half of the dissatisfied adolescents (56.0%) practiced physical activity, almost a quarter were dieting (22.9%) and more than 60% reported eating raw vegetables more frequently (Table 2).

**Table 2:** Proportions of satisfaction/dissatisfaction with weight among adolescents aged 10 to 19 years who were overweight/obese, according to health-related behaviors. ISACamp health survey, 2008/2009

Variables		n	% (95% CI)		P-value*
			Satisfied	Dissatisfied	
**Leisure physical activity**					
	Active	50	15.9 (8.4-28.0)	27.2 (19.6-36.6)	0.14
	Insufficiently active	105	58.0 (41.8-72.6)	43.4 (35.4-51.8)	
	Inactive	62	26.1 (14.7-41.9)	29.3 (21.7-38.4)	
**Fruit consumption (weekly frequency)**					
	≥ 4 times	111	58.4 (44.9-70.9)	47.0 (36.9-57.3)	0.17
	< 4 times	106	41.5 (29.1-55.1)	53.0 (42.7-63.1)	
**Raw vegetable consumption (weekly frequency)**					
	≥ 4 times	125	53.5 (42.7-64.0)	60.3 (51.6-68.5)	0.97
	< 4 times	92	46.5 (36.0-57.3)	39.7 (31.5-48.4)	
**Soft drink consumption (weekly frequency)**					
	≥ 4 times	95	49.7 (35.7-63.7)	40.3 (32.4-48.8)	0.20
	< 4 times	122	50.3 (36.3-64.3)	59.7 (51.2-67.6)	
**Do you do anything to lose weight?**					
	Yes	53	0.0	39.7 (31.9-48.1)	**< 0.01**
	No	164	100.0**	60.3 (51.9-68.1)	
**What are you doing?**					
	Dieting	12	0.0	22.9 (13.7-35.7)	a
	Exercise/sport/walking	30	0.0	56.0 (41.8-69.2)	

n = number of individuals in the unweighted sample; 95% CI = 95% confidence interval; % = percentage.

* P-value from chi-square test;

** 82 adolescents who were satisfied with their excess weight reported that they were not doing anything to lose weight;

a It was not possible to calculate the p-value because none of the satisfied adolescents were doing dieting and/or physical activity.

Lower odds regarding satisfaction with excess weight were found among older adolescents (15 to 19 years old), participants born in another town or state, those in the segments that had the highest *per capita* household income (≥ 1.5 monthly minimum wages), those with higher numbers of appliances in the household (eight or more), those who reported having one or more chronic diseases and those who reported having two or more health problems (Table 3).

**Table 3:** Proportions of satisfaction with weight among adolescents who were overweight or obese, according to sociodemographic factors and morbidities. ISACamp health survey 2008/2009

Variables		n	Satisfied % (95% CI)	P-value*	OR (95% CI)
**Sex**					
	Male	127	64.1 (54.7-72.5)	0.15	1
	Female	90	35.9 (27.5-45.3)		0.7 (0.4-1.1)
**Age (years)**					
	10 to 14	147	84.3 (74.3-90.9)	**< 0.01**	1
	15 to 19	70	15.7 (9.1-25.7)		**0.2 (0.1-0.5)**
**Place of birth**					
	Campinas	173	87.7 (76.2-94.1)	**0.03**	1
	Other town/state	44	12.3 (5.9-23.8)		**0.4 (0.2-0.9)**
***Per capita* income (monthly minimum wages)**					
	< 0.5	58	32.5 (22.8-44.0)	0.08	1
	≥ 0.5 to < 1.5	111	52.4 (41.9-62.7)		0.7 (0.4-1.5)
	≥ 1.5	48	15.0 (7.9-26.7)		**0.4 (0.2-0.9)**
**Schooling of head of household (years)**					
	0 to 7	79	32.7 (19.2-49.8)	0.51	1
	8 or more	136	67.3 (50.2-80.8)		1.3 (0.6-2.6)
**Number of appliances in the home**					
	0 to 7	42	35.8 (21.6-53.0)	**< 0.01**	1
	8 to 15	115	45.2 (31.4-59.7)		**0.2 (0.1-0.5)**
	16 or more	60	19.0 (9.6-34.1)		**0.1 (0.04-0.4)**
**Health insurance**					
	Yes	77	28.9 (18.7-41.9)	0.11	1
	No	137	71.1 (58.1-81.3)		1.8 (0.9-3.7)
**Number of chronic diseases**					
	0	175	91.3 (81.4-96.2)	**< 0.01**	1
	1 or more	39	8.7 (3.8-18.6)		**0.3 (0.1-0.7)**
**Number of health complaints**					
	0	89	53.1 (40.3-65.5)	**< 0.01**	1
	1	61	37.0 (27.7-47.5)		1.0 (0.5-2.0)
	2 or more	67	9.8 (4.8-19.0)		**0.1 (0.06-0.3)**

n = number of individuals in the unweighted sample; 95% CI = 95% confidence interval; OR = odds ratio; % = percentage.

* P-value from chi-square test.

The final logistic regression model showed lower satisfaction with weight among adolescents aged 15 to 19 years old, participants who were born outside Campinas, those who had more appliances in the household and those who mentioned having two or more health complaints (Table 4).

**Table 4:** Final logistic regression model for satisfaction with weight among overweight/obese adolescents. ISACamp health survey 2008/2009

Variables		OR	95% CI	P-value*
**Sex**				
	Male	1		
	Female	0.71	0.4-1.3	0.24
**Age (years)**				
	10 to 14	1		
	15 to 19	0.21	0.1-0.5	**< 0.01**
**Place of birth**				
	Campinas	1		
	Other town/state	0.23	0.1-0.7	**0.01**
**Number of appliances in the home**				
	0 to 7	1	1	
	8 to 15	0.14	0.05-0.4	**< 0.01**
	16 or more	0.10	0.03-0.3	**< 0.01**
**Number of health complaints**				
	0	1	1	
	1	1.18	0.5-2.8	0.71
	2 or more	0.16	0.05-0.5	**< 0.01**

OR = odds ratio; 95% CI = 95% confidence interval; % = percentage.

* P-value from chi-square test, adjusted according to all variables in this table.

## DISCUSSION

The present study found that 75.8% of the adolescents who were overweight and 24.2% who were obese were satisfied with their weight. Mäkinen et al.^13^ reported that 27.5% of their sample of adolescents presenting overweight/obesity were satisfied with their weight. Among these adolescents, 20.5% were girls (versus 79.5% who felt that they had excess weight) and 32.4% were boys (versus 66.6% who felt that they had excess weight and 1.0% who felt that they were underweight).

It was observed in the present study that younger adolescents were more satisfied with their weight. This was concordant with other studies in which teenagers with diverse weight status were evaluated.^14^^,^^15^ Concern about weight begins at the transition from adolescence to adulthood. This may be an appropriate stage for interventions focusing on health promotion and healthy eating.^5^ A qualitative study conducted among adolescents between 11 and 15 years of age who were classified as overweight or obese found that concern about weight or desire to lose weight began in the later phase of adolescence, when these individuals started to follow physical activity and dieting as strategies for weight control.^16^ Older adolescents tend to recognize that taking care of weight is a personal responsibility, implemented through making food choices and doing physical activity, especially at the time of acquiring autonomy and independence from parents.^16^

Being satisfied with body weight can prevent weight gain and binge eating. Ricciardelli et al.^17^ evaluated a group of girls for 11 years and observed that the young women who were satisfied with their bodies were 85% less likely to develop binge eating compared to those who were less satisfied.^17^ A study conducted in Minneapolis, United States, followed 376 girls who were classified as overweight or obese for five years and found that these was an association between body satisfaction and lower weight gain.^18^

In our data, females showed higher weight dissatisfaction than males: 64.1% versus 35.9%, although this difference was not statistically significant. This result agrees with the findings from a report on adolescents in the 9^th^ grade of elementary school that showed that there was twice as much body dissatisfaction (23.3%) among girls than among boys (11.6%), thus suggesting that girls were more likely to feel that they had excess weight and that boys had a greater desire to acquire a strong and muscular body.^5^

Social norms tend to value a slim body as an ideal for girls and well-defined musculature as an ideal for boys. However, this is unrealistic and unreachable for most people.^19^^,^^20^ The media and social networks disseminate the idea that slim women and muscular men represent a benchmark for success, competence and sexual attraction.^21^ On the other hand, people who are considered to be overweight or obese are usually stereotyped as lazy and careless with their own health.^22^

Lower body satisfaction can lead to adoption of unhealthy behaviors^19^ and to negative evaluation of health status.^23^ This was noted in our study, since individuals who had two or more health complaints had lower body weight satisfaction. It is likely that reverse causation may be operating.

In this study, we found that the majority of both satisfied and dissatisfied adolescents practiced some level of physical activity within the context of leisure. However, only dissatisfied adolescents did something to lose weight, which in most cases consisted of exercising. On the other hand, in a prospective study on individuals aged 9 to 14 years who were overweight or obese, the opposite was found, i.e. many of them did not practice exercise but, instead, did dieting for weight control and nonetheless gained weight.^24^ Another study showed that adolescents who were overweight, compared with those of healthy weight, presented higher prevalences of physical inactivity, eating disorders and health self-assessed as fair or poor; and lower prevalence of dating.^14^

Another study, also conducted in the United States, revealed that adolescents from 11 to 17 years old with higher purchasing power were more dissatisfied with weight.^25^ Similar data were found in the present study, through considering the number of household appliances to be a proxy for income.

One limitation of this study was its use of a single-item question to assess satisfaction with body weight, instead of a scale. This study had a cross-sectional design that provided a small-sized sample of adolescents who were classified as overweight or obese, through use of self-reported weight and height measurements. The results from validation studies on weight and height have shown that these measurements are influenced by the sociodemographic and behavioral characteristics of the age group evaluated.^26^^,^^27^ Individuals with excess weight, adolescents and females tend to underestimate weight. Furthermore, people of short stature, females and teenagers tend to overestimate height. These situations leads to errors of classification of both weight and height status.^28^^,^^29^ On the other hand, one study conducted in the city of São Paulo (which is near the city of Campinas) found that self-reported measurements showed good validity in relation to measurements that were made.^30^ We analyzed data that had been collected in 2008-2009 and which formed part of a population-based health survey and that is undertaken periodically, which allows monitoring of body weight satisfaction among adolescents.

Hardly any studies^13^^,^^14^^,^^18^ have evaluated weight satisfaction among adolescents who are overweight or obese. Hence, more are needed, in order to make important contributions towards combating this growing epidemic and improving the quality of life of this population. Measures need to be taken in order to address the duality that exists between young people who are not concerned about their weight, and therefore do not take healthy actions and may develop future health problems, and those who are overly worried about their weight and take inappropriate measures or develop eating disorders.

## CONCLUSION

This study aimed to depict the extent of satisfaction with weight among a representative sample of adolescents in a large Brazilian city. We found that more than half of these adolescents who were overweight and almost a quarter of those who were obese were satisfied with their weight. None of the adolescents who were satisfied with regard to their excess weight were following any practices aimed towards losing weight. A lower likelihood of satisfaction with body weight was noted among older adolescents (15 to 19 years), adolescents who were natives of other towns or states, those of higher socioeconomic level and those who mentioned more health complaints. These results confirm that there is a need for strategies that would promote weight control and healthy lifestyle habits.

## References

[1] Forouzanfar Afshin A, GBD 2015 Obesity Collaborators (2017). Health Effects of Overweight and Obesity in 195 Countries over 25 Years. N Engl J Med.

[2] Ng M, Fleming T, Robinson M (2014). Global, regional, and national prevalence of overweight and obesity in children and adults during 1980-2013: a systematic analysis for the Global Burden of Disease Study 2013. Lancet.

[3] Malta DC, Santos MAS, Andrade SS (2016). Tendência temporal dos indicadores de excesso de peso em adultos nas capitais brasileiras, 2006-2013 [Time trend in adult obesity indicators in Brazilian state capitals, 2006-2013]. Cien Saude Colet.

[4] Ministério do Planejamento, Orçamento e Gestão, Instituto Brasileiro de Geografia e Estatística (IBGE) (2010). Diretoria de Pesquisas Coordenação de Trabalho e Rendimento. Pesquisa de Orçamentos Familiares (POF) 2008-2009: Antropometria e Estado Nutricional de Crianças, Adolescentes e Adultos no Brasil.

[5] Ministério do Planejamento, Orçamento e Gestão, Instituto Brasileiro de Geografia e Estatística (IBGE) (2015). Diretoria de Pesquisas Coordenação de População e Indicadores Sociais. Pesquisa Nacional de Saúde do Escolar (PeNSE).

[6] Brasil. Ministério da Saúde (2011). Secretaria de Vigilância em Saúde, Departamento de Análise de Situação de Saúde. Plano de ações estratégicas para o enfrentamento das doenças crônicas não transmissíveis (DCNT) no Brasil 2011-2022.

[7] Radoszewska J (2017). The psychological determinants of obesity in children and adolescents. Dev Period Med.

[8] Del Ciampo LA, Del Ciampo IRL (2010). Adolescência e imagem corporal [Adolescence and body image]. Adolesc Saude.

[9] Santos EMC, Tassitano RM, Nascimento WMF, Petribú MMV, Cabral PC (2011). Satisfação com o peso corporal e fatores associados em estudantes do ensino médio [Body satisfaction and associated factors among high school students]. Rev Paul Pediatr.

[10] Matthiessen J, Biltoft-Jensen A, Fagt S (2014). Misperception of body weight among overweight Danish adults: trends from 1995 to 2008. Public Health Nutr.

[11] World Health Organization (2010). Global recommendations on physical activity for health.

[12] de Onis M, Onyango AW, Borghi E (2007). Development of a WHO growth reference for school-aged children and adolescents. Bull World Health Organ.

[13] Mäkinen M, Lindberg N, Komulainen E (2015). Psychological well-being in adolescents with excess weight. Nord J Psychiatry.

[14] Pelegrini A, Coqueiro R da S, Beck CC (2014). Dissatisfaction with body image among adolescent students: association with socio-demographic factors and nutritional status. Cienc Saude Colet.

[15] Kelly C, Molcho M, Nic Gabhainn S (2010). Patterns in weight reduction behaviour by weight status in schoolchildren. Public Health Nutr.

[16] Sweeting H, Smith E, Neary J, Wright C (2016). ‘Now I care’: a qualitative study of how overweight adolescents managed their weight in the transition to adulthood. BMJ Open.

[17] Ricciardelli LA, McCabe MP, Banfield S (2000). Sociocultural influences on body image and body change methods. J Adolesc Health.

[18] van den Berg P, Neumark-Sztainer D (2007). Fat'n happy 5 years later: is it bad for overweight girls to like their bodies?. J Adolesc Health.

[19] Silva ML, Taquette SR, Coutinho ES (2014). Senses of body image in adolescents in elementary school. Rev Saude Publica.

[20] Holubcikova J, Kolarcik P, Madarasova Geckova A, Van Dijk JP, Reijneveld SA (2015). Is subjective perception of negative body image among adolescents associated with bullying?. Eur J Pediatr.

[21] Lev-Ari L, Baumgarten-Katz I, Zohar AH (2014). Mirror, mirror on the wall: how women learn body dissatisfaction. Eat Behav.

[22] Martin SB, Rhea DJ, Greenleaf CA, Judd DE, Chambliss HO (2011). Weight control beliefs, body shape attitudes, and physical activity among adolescents. J Sch Health.

[23] Bispo S, Meireles AL, Côrtes MG (2013). Weight excess in adolescents in Belo Horizonte: population-based household survey. Rev Med Minas Gerais.

[24] Field AE, Austin SB, Taylor CB (2003). Relation between dieting and weight change among preadolescents and adolescents. Pediatrics.

[25] Duong HT, Roberts RE (2016). Discordance between measured weight, perceived weight, and body satisfaction among adolescents. J Psychosom Res.

[26] Silveira EA, Araújo CL, Gigante DP, Barros AJ, Lima MS (2005). Validação do peso e altura referidos para o diagnóstico do estado nutricional em uma população de adultos no Sul do Brasil [Weight and height validation for diagnosis of adult nutritional status in southern Brazil]. Cad Saude Publica.

[27] Del Duca GF, González-Chica DA, Santos JV (2012). Peso e altura autorreferidos para determinação do estado nutricional de adultos e idosos: validade e implicações em análises de dados [Self-reported weight and height for determining nutritional status of adults and elderly: validity and implications for data analysis]. Cad Saude Publica.

[28] Peixoto M do R, Benício MH, Jardim PC (2006). Validade do peso e da altura autorreferidos: o estudo de Goiânia [Validity of self-reported weight and height: the Goiânia study, Brazil]. Rev Saude Publica.

[29] Fonseca M de J, Faerstein E, Chor D, Lopes CS (2004). Validade de peso e estatura informados e índice de massa corporal: estudo pró-saúde [Validity of self-reported weight and height and the body mass index within the “Pró-saúde”]. Rev Saude Publica.

[30] Carvalho AM, Piovezan LG, Selem SSAC, Fisberg RM, Marchioni DM (2014). Validação e calibração de medidas de peso e altura autorreferidas por indivíduos da cidade de São Paulo [Validation and calibration of self-reported weight and height from individuals in the city of São Paulo]. Rev Bras Epidemiol.

